# The predictive ability of blood-based biomarkers to detect bacteremia in hospitalized neonatal foals

**DOI:** 10.1016/j.tvjl.2025.106427

**Published:** 2025-09-02

**Authors:** Amanda N. Samuels, Niamh M. Collins, Kelly Hanlon, Celine Bartish, Payton Kelly, Ahmed M. Kamr, Ramiro E. Toribio

**Affiliations:** a Department of Veterinary Clinical Sciences, College of Veterinary Medicine, The Ohio State University, Columbus, USA; b Clovelly Intensive Care Unit, Scone Equine Hospital, Scone, New South Wales, Australia

**Keywords:** Bacteremia, Equine neonate, Sepsis, Hematology, Immunology, Biomarkers

## Abstract

Early and accurate identification of septicemia in neonatal foals improves survival. In human medicine, the neutrophil-to-lymphocyte ratio (NLR), neutrophil-to-monocyte ratio (NMR), monocyte-to-lymphocyte ratio (MLR), and plasma cell-free DNA (cfDNA) aid in early bacteremia detection. This study evaluated the diagnostic utility of these markers in conjunction with other clinical and hematological parameters in hospitalized foals < 5 days old to predict positive blood culture at admission and to distinguish between Gram-positive, Gram-negative, or polymicrobial bacteremia. A total of 391 foals with a complete blood count and aseptically obtained blood culture at admission were included. Physical exam and hematologic parameters, including white blood cell count (WBC) and immunoglobulin G (IgG), were incorporated into logistic regression models, with the area under the curve (AUC) used to assess predictive performance. Plasma cfDNA was measured via fluorometry. We found that plasma cfDNA, IgG, NLR, and WBC were independent predictors of bacteremia, and a composite model demonstrated excellent discriminatory ability to identify foals with a positive blood culture (AUC = 0.806). Additionally, plasma cfDNA, IgG, and neutrophil counts were independent predictors of Gram-negative bacteremia, and a composite model demonstrated excellent discrimination (AUC = 0.807), and monocyte count and age predicted Gram-positive bacteremia with a composite model that demonstrated fair discriminatory ability (AUC = 0.67). Our findings demonstrate that NLR and plasma cfDNA are significantly altered in bacteremic foals. whereas the NMR and MLR do not differ significantly between groups. Combining these markers with other clinicopathologic variables may enable early identification and timely intervention in affected foals.

## Introduction

Bacterial infections are a significant cause of morbidity and mortality in neonatal foals (Bookbinder et al., 2023; Cohen, 1994; Corley et al., 2007; Hytychová, Bezděková, 2015; Koterba et al., 1984; Sanchez et al., 2008). Identifying bloodstream infections early during hospitalization and initiating the appropriate treatment improves survival outcomes in human medicine (Liu et al., 2017; Rello et al., 2017). While similar data in foals is limited, early and accurate identification of bloodstream infections are likely to improve survival rates significantly (Bookbinder et al., 2023; Theelen et al., 2019). Being able to further distinguish between Gram-positive, Gram-negative, or polymicrobial bloodstream infections could enable targeted antimicrobial therapy, enhance antimicrobial stewardship, and provide prognostic information since the likelihood of survival varies depending on the infection type (Barton et al., 1998; Corley et al., 2007; Stewart et al., 2002; Theelen et al., 2019).

Definitive diagnosis of bloodstream infections requires isolation of the bacteria from a blood culture, which often requires 48–72 h and has suboptimal sensitivity (Hackett et al., 2015; Russell et al., 2008). The equine-specific sepsis scoring system is intended to raise suspicion of sepsis in neonatal foals (Brewer, Koterba, 1988; Wong et al., 2018), but its clinical utility depends upon historical and clinicopathologic information that is not always available. Furthermore, correct identification of bacteremic foals using the sepsis scoring system is variable (Furr, McKenzie, 2020; Stewart et al., 2002). Specifically, the calculated sensitivity and specificity to detect bacteremia using the sepsis scoring system varies from 54 % to 62 % and 66 % to 73 %, respectively (Furr, McKenzie, 2020; Weber et al., 2015; Wong et al., 2018). Biomarkers have been investigated in foals with the goal of early and accurate diagnosis of sepsis and bloodstream infections. Hyperlactatemia, increased serum amyloid A concentration, rectal temperature alteration (hypothermia or hyperthermia), hypoglycemia, and neutropenia have been associated with sepsis and bacteremia in foals, but these biomarkers are nonspecific, and their reliability across different studies is inconsistent (Barr, Nieman, 2022; Borchers et al., 2013; Corley et al., 2007; Furr, McKenzie, 2020; Hoeberg et al., 2022; Koterba et al., 1984; Wilkins et al., 2025)

In human medicine, the neutrophil-to-lymphocyte ratio (NLR), neutrophil-to-monocyte ratio (NMR), monocyte-to-lymphocyte ratio (MLR), and plasma cell-free (cfDNA) concentrations are used to assess inflammatory status and immune function (Li et al., 2020, 2024; Ng et al., 2022; Xia et al., 2022; Zahorec, 2021). Specifically, the NLR has improved diagnostic accuracy over other biomarkers to detect bacteremia in patients admitted to the emergency room (Ljungström et al., 2017; PC de Jager et al., 2010). The NMR is an independent risk factor for human neonatal sepsis, with a higher NMR in non-survivors (Xia et al., 2022). The MLR is significantly higher in patients hospitalized for fever due to bacterial infection (Naess et al., 2017) and in patients with Gram-negative blood cultures than in those with Gram-positive blood cultures (Djordjevic et al., 2018). The plasma cfDNA concentrations are consistently increased in septic and bacteremic human patients (Charoensappakit et al., 2023; Huttunen et al., 2011; Urosevic et al., 2022). The NLR, MNR, and MLR can be readily obtained from a peripheral complete blood count (CBC), and cfDNA can be measured within hours using commercially available cfDNA isolation kits and the Qubit^™^ fluorometer, a commercially available tabletop fluorometric device.

There is an unmet need for more prompt and accurate diagnostic biomarkers to identify bacteremic foals at admission. The NLR correlates with disease severity and non-survival in hospitalized neonatal foals, but its ability to detect bacteremia has been underexplored (Samuels et al., 2024). cfDNA concentrations are increased in the serum of horses with equine recurrent uveitis, in the synovial fluid of horses with experimentally induced osteoarthritis, and in the plasma of horses with colic, including those with inflammatory lesions; however, its value in identifying septic foals remains equivocal, and its ability to detect bacteremia in foals remains unclear due to methodological limitations, small sample size, and patient classification criteria (Bayless et al., 2022, 2024; Colmer et al., 2021; Fingerhut et al., 2019; Hobbs et al., 2024; Panizzi et al., 2023). To the authors’ knowledge, the NMR and MLR have not been investigated in neonatal foals.

We propose that the NLR NMR, MLR, and plasma cfDNA at admission may have diagnostic utility for identifying bacteremia in foals less than five days of age that present to tertiary equine referral hospitals. The objective of this study was to determine the utility of the NLR, NMR, MLR, and plasma cfDNA to predict the presence of bacteremia, and to evaluate the diagnostic performance of these hematological parameters to discriminate between Gram-positive, Gram-negative, and polymicrobial bacterial bloodstream infections. We hypothesize that these hematological parameters will be associated with bacteremia in our patient population and will demonstrate diagnostic utility in identifying bacteremic foals, as well as distinguishing between Gram-positive, Gram-negative, and polymicrobial bacteremia. Continuing to elucidate hematological parameters that can identify bacteremic foals early during hospitalization will provide meaningful information that will impact clinicians’ decisions regarding case management and communication with owners.

## Materials and Methods

### Animal and inclusion criteria

A total of 391 foals ≤ 5 days of age of any breed and sex from 2018 to 2024 foaling seasons were included. Hospitalized foals were admitted to two equine referral hospitals (The Ohio State University [OSU] Equine Center, Columbus, U.S.A., and Scone Equine Hospital, Scone, Australia). Foals were included in this study if they had (1) a CBC and a blood culture obtained aseptically at admission; (2) documentation of either discharge from the hospital or non-survival (death or euthanasia); (3) final blood culture results available; (4) were hospitalized as the primary patient (i.e., not as a healthy companion animal or presenting solely for a wellness examination).

### Medical records review

Information obtained from the foals’ medical records at admission included breed, sex, age, physical exam parameters (rectal temperature, respiratory rate, and heart rate), clinicopathologic data (CBC, blood L-lactate and glucose concentrations, packed cell volume [PCV], and total solids [TS]), serum immunoglobulin G (IgG) concentrations, and organisms isolated from blood cultures. Diagnostic testing was at the clinicians’ discretion; therefore, outside of the inclusion criteria, not all information was collected on each foal.

Each institution processed blood samples for bacterial culture with slight variations in blood culture methods. Generally, 10–20 mL of blood for culture was collected using aseptic techniques from the jugular or cephalic vein or through a jugular catheter immediately after catheter placement. The blood was sterilely transferred to a commercial blood culture bottle (e.g., tryptone-soy broth containing sodium polyanethol sulfonate) and incubated aerobically at 36°C (± 1°C) for a maximum of 7 days and observed daily. Bacterial colonies grown on media were identified using standard microbiological methods. Information for each foal was retrieved from finalized reports confirming the bacterial organism isolated from the blood cultures and the number of isolates if more than one microorganism was identified. The blood culture results were defined as polymicrobial when more than one bacterial organism was obtained from the same blood culture. At the OSU Equine Center, serum IgG concentrations were measured by turbidimetry using an automated system (Roche COBAS c501) and CBC analyzed with an ADVIA hematology system (Siemens Medical Solutions, Malvern, PA), while at the Scone Equine Hospital, serum IgG concentrations were determined with the DVM Rapid Test II (Value Diagnostics, MAI Animal Health, Elmwood, WI) and the CBC with the Beckman Coulter AcT Diff (Beckman Coulter Life Sciences, Indianapolis, IN). Both the ADVIA hematology system and the Beckman Coulter AcT Diff provide absolute leukocyte differentials, which were used to calculate hematological ratios (e.g., NLR, MLR).

Foals were further evaluated based on the equine neonatal systemic inflammatory response syndrome (SIRS) criteria, including rectal temperature (> 39.2°C or < 37.2°C), heart rate (HR) (>120 beats/min for foals 0 – 72 hr and > 120 beats/min for foals > 72 hr), respiratory rate (>56 breaths/min), white blood cell (WBC) counts (> 14.4 ×109/L or < 6.9 ×109/L for foals 0 – 72 hr and > 12.5 ×109/L or < 4.0 ×109/L for foals > 72 hr), blood L-lactate (> 5 mmol/L for foals 0 – 72 hr and > 2.5 mmol/L for foals > 72 hr), and blood glucose concentrations (< 50 mg/dL) (Wong et al., 2018). To meet the equine neonatal SIRS criteria, foals were required to meet at least three criteria, one of which had to be either an abnormal rectal temperature or white blood cell count. SIRS status was reported as a binary variable (yes/no), rather than by tallying individual SIRS scores.

Prematurity was defined based on gestational age, where < 320 days of gestation indicates prematurity. Survivors were foals discharged alive, and non-survivors were foals that died or were euthanized either due to a grave prognosis or for medical reasons.

### Sample collection and cfDNA measurements

Plasma cfDNA concentrations were measured in a subset of foals (n = 83), representing all eligible cases during the study period with both plasma and blood culture results available. However, a post hoc power analysis using the observed effect size (r = 0.32), alpha level of 0.05, and sample size of 83 foals indicated an estimated power of 80 %. Of those, 59 % (49/83) had a negative blood culture and 41 % (34/83) had a positive blood culture. Blood samples were obtained from an intravenous jugular catheter placed sterilely at admission and collected into K2-EDTA tubes. After centrifugation at 3000 g for 10 min at room temperature, plasma was harvested and frozen at −80°C until processing. The cfDNA was extracted from thawed plasma using a commercially available kit according to the manufacturer’s instructions (Cell-Free DNA Purification Kit, Active Motif, Carlsbad, CA, USA). Plasma cfDNA quantification was performed in duplicate using a benchtop fluorometer (Qubit 4 fluorometer, Thermo Fisher Scientific, Waltham, MA, USA) and associated reagents (Qubit 1x dsDNA HS Assay Kit and Qubit Assay Tubes, Thermo Fisher Scientific) as per the manufacturer’s instructions. The average cfDNA values were used for analysis. Dilutional linearity for this assay was obtained with serial dilutions (1:2, 1:4, 1:10) of plasma samples with known cfDNA concentration. Linear regression analysis demonstrated an average R^2^ value of 0.99 for two independent sample sets. To assess intra-assay precision, cfDNA concentrations were measured in four independent plasma samples in triplicate, and the coefficient of variation (CV%) was calculated. The observed CVs were 1.17 %, 0.86 %, 3.12 %, and 0.45 %.

### Statistics

Data were tested for normality by the Shapiro-Wilk statistics and were not normally distributed. Thus, values are presented as median and interquartile range (IQR). Univariate analysis for categorical and continuous variables were performed using Pearson’s Chi-square test and Fisher’s exact test or Mann-Whitney U test, respectively. Variables with P < 0.1 in univariate analysis were included in multivariate analysis; thus, each model was generated using a unique set of risk factors. Logistic regression analysis with backward stepwise elimination was used to assess independent predictors for bacteremia, and the Hosmer-Lemeshow test was used to ensure the goodness of fit of statistical models. The *a priori* significance level required for a variable to be retained in the final model was *P* < 0.1 unless otherwise stated. Comparisons of the prognostic and diagnostic accuracy of significant variables were performed using receiver operating characteristics (ROC) curves. The area under the curve (AUC) analysis was calculated ranging from 0.5 to 1.0. The Youden index was used to determine the optimal specificity and sensitivity cutoffs. Statistical analyses were performed using IBM SPSS Statistics 28.0 (IBM Corporation, Armonk, NY, USA) and Prism 9.0 (GraphPad Software, Inc., La Jolla, CA, USA). Figures were created using Prism 9.0 (GraphPad Software, Inc., La Jolla, CA, USA) and Inkscape (Inkscape Project, https://inkscape.org). Significance was set at P < 0.05 unless otherwise stated.

## Results

### Study sample demographics

A total of 391 foals were included in the analysis and categorized into two groups based on blood culture status. Of these, 135 foals (35 %; 135/391) had a positive blood culture, while 256 foals (65 %; 256/391) had a negative blood culture. Foals with a positive blood culture were further subdivided into three groups based on the classification of bacteria isolated: Gram-positive (41 %, n = 56/135), Gram-negative bacteria (46 %, n = 62/135), or polymicrobial (13 %, n = 17/135). The isolated organisms are listed in Supplementary Table 1.

Among the 391 foals, 58 % were colts (226/391) and 42 % were fillies (165/391). Of the foals with a positive blood culture, 60 % were colts (81/135) and 40 % were fillies (54/135), and of the foals with a negative blood culture, 57 % were colts (145/256) and 43 % were fillies (111/256). No association was found between sex and blood culture status (Pearson Chi-Square = 0.509, df = 1, *p* = 0.476), and a Fisher’s Exact Test confirmed the absence of a significant association (*p* = 0.519).

The median age of 391 foals was 24 hr (IQR: 8–48). In the cohort of 391 foals, there was no correlation between age and the NLR (ρ = 0.035, 95 % CI: −0.067–0.137, *p* = 0.50), plasma cfDNA concentration (ρ = 0.003, 95 % CI: −0.220–0.219, *p* = 0.97), or neutrophil count (ρ = −0.090, 95 % CI: −0.191–0.012, *p* = 0.08). However, a significant negative correlation was found between age and lymphocyte count (ρ = −0.255, 95 % CI: −0.348 to −0.158, *p* < 0.0001). Of the foals with a negative blood culture, 70 % were ≤ 24 h old (181/256), 16 % were > 24 h but < 72 h old (40/256), and 14 % were ≥ 72 h old but ≤ 120 hr (35/256). In the cohort of negative blood culture foals, there was no correlation between age and the NLR (ρ = 0.120, 95 % CI: −0.006–0.241, *p* = 0.06), plasma cfDNA concentration (ρ = 0.005, 95 % CI: −0.293–0.2184, *p* = 0.97), or neutrophil count (ρ = −0.029, 95 % CI: −0.154–0.097, *p* = 0.64). However, a significant negative correlation was found between age and lymphocyte count (ρ = −0.260, 95 % CI: −0.374 to −0.138, *p* < 0.0001). Of the foals with a positive blood culture, 52 % were ≤ 24 h old (70/135), 28 % were > 24 h but < 72 h old (38/135), and 20 % were ≥ 72 h old but ≤ 120 hr (27/135). In this cohort of foals with a positive blood culture, there was no correlation between age and the NLR (ρ = 0.012, 95 % CI: −0.161–0.184, *p* = 0.88), plasma cfDNA concentration (ρ = 0.004, 95 % CI: −0.356–0.349, *p* = 0.98), neutrophil counts (ρ = −0.042, 95 % CI: −0.213–0.313, *p* = 0.62), or lymphocyte count (ρ = −0.156, 95 % CI: −0.320–0.017, *p* = 0.07).

Maturity status was recorded in 59 % (231/391) of foals, and 14 % (31/231) of these foals were identified as premature. Of the premature foals, 58 % (18/31) had a positive blood culture and 42 % (13/31) had a negative blood culture. No association was found between prematurity and blood culture status (Pearson Chi-Square = 0.837, df = 1, *p* = 0.360), and a Fisher’s Exact Test confirmed the absence of a significant association (*p* = 0.452).

Among the 391 foals, 152 were determined to fit the equine neonatal SIRS criteria (39 %, 152/391). Of these SIRS foals, 45 % (69/152) had a positive blood culture and 54 % (83/152) had a negative blood culture. SIRS foals with a positive blood culture were categorized into three groups: 38 % (26/69) had Gram-positive, 52 % (36/69) had Gram-negative, and 10 % (7/69) had polymicrobial infections. In this cohort, blood culture status was not significantly associated with prematurity (Pearson Chi-Square = 1.606, df = 1, *p* = 0.205) or sex (Pearson Chi-Square = 0.563, df = 1, *p* = 0.453). A Fisher’s Exact Test confirmed the absence of a significant association (p = 0.236 and *p* = 0.510, respectively). However, there was a significant association between SIRS criteria and positive blood culture (Pearson Chi-Square = 5.44, df = 1, *p* = 0.02). Fisher’s Exact Test confirmed the significant relationship (*p* = 0.02).

Breeds represented in the total foal cohort included Thoroughbred (n = 165), Standardbred (n = 98), Quarter Horse (n = 56), Friesian (n = 15), Dutch Warmblood (n = 9), Belgian (n = 8), American Saddlebred (n = 6), Gypsy Vanner (n = 5), American Miniature Horse (n = 5), Arabian (n = 5), Percheron (n = 4), Rocky Mountain Horse (n = 3), American Paint (n = 3), Haflinger (n = 3), Morgan (n = 3), Clydesdale (n = 2), and Shire (n = 1).

### Blood culture status and non-survival

Of the 391 hospitalized foals in this study, 19 % (73/391) did not survive. Among the non-survivors, 40 % (29/73) had a positive blood culture and 60 % (44/73) had a negative blood culture. Foals with polymicrobial infections had the highest non-survival rate (7/17, 41 %), followed by those with Gram-negative infections (18/62, 29 %) and Gram-positive infections (4/56, 7 %). Survival rates differed significantly among these groups (Pearson Chi-Square = 12.8, df = 2, p = 0.002).

The odds ratio (OR) of non-survival for foals with a positive blood culture was 1.25 (95 % CI: 0.74–2.10), but this association was not statistically significant (*p* = 0.402). The OR for non-survival in foals with Gram-positive infections was 0.286 (95 % CI: 0.10–0.818, *p* = 0.01). In contrast, foals with Gram-negative and polymicrobial infections had significantly higher odds of non-survival, with ORs of 1.95 (95 % CI: 1.05–3.62, *p* = 0.03) and 3.15 (95 % CI: 1.16–8.57, *p* = 0.02), respectively.

### Performance of clinical and hematological parameters to predict positive blood culture in hospitalized foals

Comparative clinical and hematologic results for foals with negative and positive blood cultures are presented in Table 1. Univariate analysis identified several parameters significantly associated with a positive blood culture, including age, HR, TS, plasma cfDNA, WBC count, IgG concentrations, lymphocyte count, neutrophil count, monocyte count, and the NLR.

Multivariable logistic regression was conducted using all 391 foals. Neutrophil and lymphocyte counts were excluded from this model due to redundancy with the NLR, which was retained as a more biologically relevant and independent predictor. Tolerance and variance inflation factor (VIF) values confirmed no collinearity between WBC and NLR (tolerance = 0.831; VIF = 1.20). The four remaining independent predictors—cfDNA, IgG, NLR, and WBC—formed **Model 1** (Table 2).

To assess the utility of readily and rapidly available hematological variables, a second model (**Model 2**) excluded cfDNA. In this analysis, age, IgG, TS, and WBC emerged as independent predictors of positive blood culture status and the composite was defined as **Model 2** (Table 2).

**Fig. 1.**
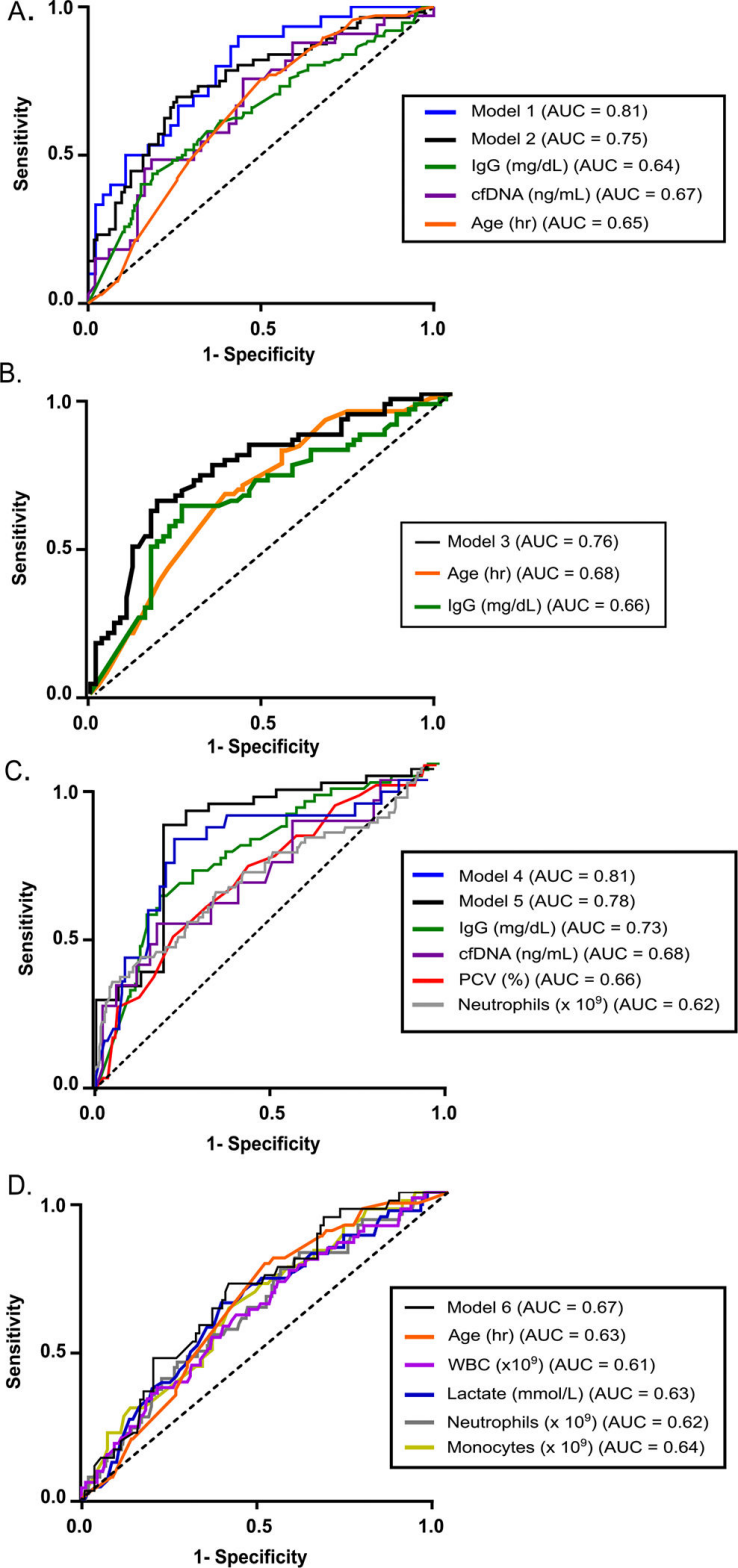
Receiver operating characteristic curves of parameters that were predictors of a positive blood culture in all hospitalized foals (A), a subset of SIRS foals (B) and clinicopathologic predictors for specifically Gram-negative bacteremia in all hospitalized foals (C), and Gram-positive bacteremia in all hospitalized foals (D).

To further evaluate the predictive value of risk factors identified in the univariate analysis, ROC curve analysis was performed, and the AUC was calculated for parameters significantly associated with a positive blood culture (NLR, WBC, IgG, cfDNA, age, TS, monocytes, neutrophils, and lymphocytes), as well as for Model 1 and Model 2 (Table 3). Fig. 1A displays the ROC curves for predictors with an AUC > 0.64 to highlight those with modest or greater discriminatory ability.

### Performance of clinical and hematological parameters to predict a positive blood culture in foals meeting equine neonatal SIRS criteria

We evaluated whether specific clinical and hematological parameters could help distinguish between positive and negative blood cultures in foals meeting the equine neonatal SIRS criteria. Comparative results for these foals are shown in Table 4.

Univariate analysis identified age, cfDNA, HR, and IgG concentrations as significantly associated with a positive blood culture in this subset of foals meeting the equine neonatal SIRS criteria. In this cohort, cfDNA was measured in a total of 40 foals, with 55 % having a negative blood culture (22/40) and 45 % (18/40) having a positive blood culture. Multivariable analysis identified age and IgG as independent predictors—these variables composed **Model 3**. (Table 2).

ROC curve analysis was performed, and AUC was calculated on the identified independent predictors (IgG and age) as well as for Model 3 (Fig. 1B, Table 3).

### Performance of clinical and hematological parameters to predict foals with Gram-negative, Gram-positive, or polymicrobial blood culture

Clinical and hematologic results for these foals are shown in Table 1. Univariate analysis identified HR, plasma cfDNA, blood glucose, and IgG concentrations, PCV, WBC counts, lymphocyte counts, and neutrophil counts as significantly associated with Gram-negative blood culture status.

Multivariable analysis identified cfDNA, neutrophils, and IgG as independent predictors—this composite model was defined as **Model 4**. Excluding cfDNA to evaluate parameters readily and easily available in clinics led to **Model 5**, with IgG and PCV as predictors of Gram-negative blood culture (Table 2). ROC curve analysis was performed, and the AUC was calculated for the identified independent predictors (IgG, neutrophil counts, plasma cfDNA concentration, and PCV), as well as for Model 4 and Model 5 (Fig. 1C, Table 3).

Comparative clinical and hematologic results of foals with a Gram-positive blood culture are presented in Table 1. Univariate predictors included age, WBC, L-lactate, neutrophils, and monocytes. Multivariable analysis identified age and as independent predictors and this composite model was defined as **Model 6** (Table 2). ROC curve analysis was performed, and AUC values were calculated for the independent predictors (age, WBC counts, blood L-lactate concentration, neutrophil count, monocyte count) and Model 6 (Fig. 1D and Table 3).

Comparative clinical and hematologic results of foals with polymicrobial bacterial cultures are provided in Table 1. Univariate predictors included age, NLR, and IgG. While all three were retained in multivariable analysis as independent predictors, the overall model showed poor fit (Hosmer–Lemeshow chi-square = 171, df = 8, p < 0.001), and thus no numbered model was assigned. ROC curve analysis was conducted, and AUC values were calculated on age, NLR, and IgG concentration (Table 3).

## Discussion

This study leveraged a large, multicenter cohort of 391 hospitalized foals ≤ 5 days of age to evaluate clinical and hematological parameters associated with bloodstream infections. By applying univariate and multivariable logistic regression analyses, we identified predictors that could aid in the early recognition of bacteremia. This study identified significant differences in age, heart rate (HR), WBC count, neutrophil, lymphocyte, and monocyte counts, NLR, IgG, total solids (TS), and cfDNA between bacteremic and non-bacteremic foals. A composite model incorporating cfDNA, IgG, NLR, and WBC count demonstrated good diagnostic ability to identify bacteremic foals at presentation. In the subset of foals meeting the SIRS criteria (n = 152), age, HR, respiratory rate, WBC count, neutrophil and lymphocyte counts, IgG, L-lactate, and cfDNA were significantly different between groups, with a composite model of IgG and age providing the best discriminatory performance. Although NLR NMR, and MLR differed in bacteremic SIRS foals, these differences were not statistically significant. Additionally, clinical and hematological parameters differed significantly among foals with Gram-negative, Gram-positive, and polymicrobial bacteremia, and composite models for Gram-negative and Gram-positive infections demonstrated adequate to good discriminatory ability to identify these foals at presentation.

This study showed that bacteremic foals had a lower NLR compared to those with a negative blood culture, and the NLR was an independent predictor for bacteremia. However, the NLR as a single parameter had poor discriminatory power to distinguish between bacteremic and non-bacteremic foals. In the subset of foals meeting the equine neonatal SIRS criteria, the NLR was lower in blood culture-positive foals compared to those with a negative blood culture, although the difference was not statistically significant. It is possible that the smaller number of foals in this cohort precluded the ability to reach statistical significance. Additionally, the NLR may vary depending on the nature of systemic inflammation and the underlying condition. In human medicine, the NLR can adequately differentiate between individuals with bacteremia and those who are not bacteremic in the emergency and ICU setting (S. Liu et al., 2021; PC de Jager et al., 2010). Other reports reveal that the predictive ability of the NLR may depend upon the underlying condition, as one report found no discriminatory ability in human patients with peritonitis but good predictive ability in those with concurrent pancreatitis (Djordjevic et al., 2018). We did not stratify foals into underlying conditions, and the predictive ability of the NLR in foals might depend upon underlying conditions. Because underlying diagnoses were not classified in this study, the influence of disease type on the NLR’s performance in foals remains unclear and warrants further investigation. We found, however, that the diagnostic performance of the NLR was significantly improved when combined with serum IgG and plasma cfDNA concentrations and WBC counts. Therefore, the combination of these parameters may help a clinician decide which hospitalized foals have a greater likelihood of being bacteremic and thus should be treated aggressively.

The NMR and MLR, and their association with infection in equids, had not been investigated prior to this study. The NMR and MLR are hypothesized to provide more insight into the balance of immune cell populations, potentially providing a more integrated measure of systemic inflammation than absolute cell counts alone. Unlike in human medicine, we found that the NMR and MLR did not differ based on blood culture status. While the NMR and MLR were not significant for distinguishing bacteremia in foals in this study, these ratios may still hold diagnostic or prognostic value in other equine populations with different types or severities of systemic disease. Future research should explore their utility across broader clinical contexts. Monocyte count were significantly lower in bacteremic foals, particularly among those with a Gram-positive organism isolated. Monocyte counts emerged as a significant predictor of positive blood cultures and demonstrated moderate discriminative power in predicting Gram-positive infections in the hospitalized foals of this study. Literature regarding monocyte counts in hospitalized foals is scarce, and some studies in human medicine report an association between decreased monocyte counts, sepsis, and bacteremia (Chung et al., 2019; Saenz et al., 2001). Monocyte count was the most reliable predictor of bacteremia in children who had already developed neutropenia and fever (Rackoff et al., 1996). More work is required to further elucidate the role of monocytes in hospitalized foals.

In our study, increased plasma cfDNA concentration was significantly associated with bacteremia and, as a single parameter, could adequately differentiate bacteremic from non-bacteremic foals (AUC = 0.67). Further, in a subset of SIRS foals, cfDNA was increased in bacteremic foals compared to non-bacteremic foals. This result differs from previous studies, which found no difference in plasma cfDNA between septic, sick non-septic, and healthy foals (Hobbs et al., 2024). Our data, however, aligns with other studies that consistently found increased concentrations of plasma cfDNA in dogs (Letendre and Goggs, 2017), humans (Charoensappakit et al., 2023; Lenz et al., 2022a; Urosevic et al., 2022), and adult horses with signs of systemic inflammation (Bayless et al., 2024). Possible reasons for this discrepancy include the larger sample size in this study, which may have contributed to achieving statistical significance. Additionally, foals in our study were classified based on a definitive diagnosis of bacteremia, confirmed by a positive blood culture collected at the time of admission, rather than on sepsis score classification. Our study also found that plasma cfDNA concentration was significantly associated with Gram-negative blood cultures and served as an independent predictor, suggesting that plasma cfDNA could be useful in identifying foals with Gram-negative bacteremia. Our study did not assess DNase or nuclease activity, which can influence circulating cfDNA levels. Altered DNase activity has been reported in disease states and could contribute to variability in cfDNA concentrations (Golonka et al., 2018; Lenz et al., 2022). That said, we were still able to demonstrate that bacteremic foals, including those with SIRS, have increased plasma levels of cfDNA. Future investigations should consider measuring nuclease activity to better understand its impact on cfDNA dynamics and its utility as a biomarker in equine bacteremia. Nonetheless, this data adds to the growing body of literature characterizing the significance of plasma cfDNA in equine medicine.

Beyond investigating the role of the NLR NMR, MLR, and plasma cfDNA concentrations, our study identified other significant clinical and hematological parameters that are useful in the early identification of bacteremic foals. Age is a critical factor in neonatal immunity and disease susceptibility, and we found a significant age difference between foals with and without bacteremia, and age demonstrated moderate predictive ability (AUC = 0.65) as an independent variable and was retained in multiple logistic regression models. This finding parallels a similar finding in a recently published manuscript that found age to be significantly different between foals with and without an infection (Wilkins et al., 2025). However, age did not significantly correlate with NLR, neutrophil counts, or cfDNA concentrations, suggesting that age may influence other physiological or hematologic parameters such as white blood cell function. Specifically, foals < 21 days exhibit reduced neutrophil killing capacity, and those under 5 days have altered cytokine responses (Demmers et al., 2001; Wagner et al., 2010). Interestingly, in this study, there was a negative correlation between lymphocyte count and age in foals with a negative blood culture. The reason for this is unclear, but these age-related factors highlight the complexity of host-pathogen interactions in neonatal foals and underscore the importance of considering age as a biological variable. The significance of age in our study and others (Wilkins et al. 2025) supports the need for future investigations to use narrower age ranges to better assess biomarker performance and disease risk.

In our study, low serum IgG concentration was consistently identified as an independent predictor and was retained in all the composite models except Model 6. The retention of IgG in most models suggests it is a critical protective factor against bacterial invasion and neonatal disease. Decreased serum IgG concentration impairs the ability to mount an appropriate immune response to infections, and coupled with other systemic and hematological disturbances, likely increases the risk of bacteremia. In this study, decreased serum IgG concentration was associated with bacteremia, specifically Gram-negative and polymicrobial blood cultures, but not with Gram-positive blood cultures. This highlights the complex relationship between IgG concentrations, bacteremia, and sepsis. While some studies have shown no association between IgG concentration at admission and blood culture status (Furr, McKenzie, 2020; Hollis et al., 2008; Stewart et al., 2002), others have demonstrated that low IgG concentrations increase the risk of sepsis development (Hytychová and Bezděková, 2015; Robinson et al., 1993). These variations might be influenced by study design and population characteristics, but also suggest differences may relate to specific bacterial organisms involved.

WBC and neutrophil counts are commonly associated with sepsis in neonatal foals (Koterba et al., 1984; Gayle et al., 1998; Samuels et al., 2024; Scalco et al., 2023; Wong et al., 2018). In this study, leukopenia was associated with bacteremia, although it was not specifically linked to Gram-negative, Gram-positive, or polymicrobial infections. WBC counts were included in predictive models identifying bacteremic foals, differing from prior studies that did not find WBC counts predictive of bacteremia (Furr, McKenzie, 2020). This discrepancy may stem from differences in foal age, sample size, and the clinical parameters used in dent predictor of positive blood culture, including both Gram-negative and Gram-positive infections, and was specifically retained in models predicting Gram-negative bacterial isolates. This finding aligns with previous studies emphasizing the role of neutrophil counts in identifying foals with Gram-negative infections (Corley et al., 2007; Stewart et al., 2002) and in a newer study where toxic neutrophils retained the predictive ability to identify foals with an infection (Wilkins et al., 2025). Due to the retrospective nature of this study, toxic changes of neutrophils were not assessed as an independent variable.

In addition to generating predictive models for early identification of bacteremia in foals, our results highlight distinct clinical and hematologic responses to Gram-negative, Gram-positive, and polymicrobial infections, suggesting that the foal’s immune response varies according to the type of microorganism involved. Studies in humans (Draing et al., 2008; Skovbjerg et al., 2010) and adult equids (DeClue et al., 2012) have demonstrated that Gram-positive and Gram-negative bacteria induce different cytokine profiles; however, the immunological response in foals to different microorganisms has not been thoroughly investigated. Our results further support this differential immune response since each of our models included a distinct set of predictors. Interestingly, the model with the strongest predictive ability was model 4 (AUC = 0.807), which predicted foals with Gram-negative bacterial isolates. Foals with Gram-negative bacteremia may experience greater systemic and clinical disturbances due to circulating endotoxins, which could result in more distinct patterns among the predictors to enhance model performance. In our study, foals with polymicrobial blood cultures had the highest rate of non-survival, but we were unable to generate a statistically significant model to identify these foals at admission. The small sample size of this cohort of foals likely limited the ability to detect significant relationships between predictors and polymicrobial bacteremia.

Repeat blood cultures were not routinely collected in all hospitalized foals, and cultures obtained during hospitalization were not analyzed in our study. Relying on a single blood culture at admission inherently carries the risk of false negatives. Some foals may have been bacteremic at admission but did not yield a positive culture due to intermittent bacteremia, volume of blood collected, prior antimicrobial treatment, or technical issues. Therefore, it is likely that some truly septic foals were misclassified as non-bacteremic in our study, which may have impacted the observed associations. Future studies employing serial blood cultures or blood cultures from more than one site could improve the ability to detect these foals. However, including foals that later developed bacteremia would have made it difficult to distinguish between those who were bacteremic at admission and those who acquired bacteremia later during their hospitalization due to changes in their clinical status. Our models provide adequate predictive ability to identify foals at admission, which could prompt early and aggressive treatment and facilitate communication with the client.

The overall foal survival rate in this study was higher (81 %) compared to some studies (Barton et al., 1998; Corley et al., 2007; Gayle et al., 1998; Giguère et al., 2017; Koterba et al., 1984; Raisis et al., 1996; Sanchez et al., 2008; Stewart et al., 2002; Theelen et al., 2019) although comparable to recently published studies (Furr, McKenzie, 2020; Hoeberg et al., 2022; Scalco et al., 2023). There was a significant difference in survival rates among foals with Gram-positive (93 %), Gram-negative (71 %), and polymicrobial (59 %) blood cultures, with Gram-negative and polymicrobial infections being associated with increased odds of non-survival. This finding contrasts with previous studies where the type of infection did not affect the survival rate (Corley et al., 2007; Gayle et al., 1998; Hytychová, Bezděková, 2015; Russell et al., 2008); however, our results align with previous studies where Gram-negative and polymicrobial blood cultures increased the rate of non-survival (Barton et al., 1998; Stewart et al., 2002; Theelen et al., 2019). Direct comparisons are difficult due to differences in the foal populations (e.g., age, prior treatments), owners’ financial situations, varying management strategies across clinics, and geography. Nevertheless, our results suggest that survival rates likely depend upon the patient population and bacterial organisms. Antimicrobial susceptibility was not evaluated in this study, and it is possible that changing antimicrobial resistance profiles could influence response to therapy and subsequent survival.

A limitation of this study is the absence of a healthy control group, which may restrict interpretation of how hematological parameters differ from baseline values; however, the study was intentionally designed to evaluate the diagnostic performance of these markers to distinguish bacteremia within a population of sick, hospitalized foals, where clinical decision-making is most relevant. Additionally, our study did not control for treatment and medications received before arrival, which could influence the blood culture status and the immune response. Due to the sample size, our study did not examine the impact of different bacterial species on clinical and hematological parameters. Lastly, we did not stratify foals based on underlying disease processes, which may have led to the identification of additional relationships between measured parameters and blood culture status. A larger prospective study is warranted to explore the effects of previous treatments, different bacterial species, and underlying disease processes. Additionally, these models have not been clinically validated and should not be extrapolated to foals ≥ 5 days. It should also be noted that quantification of plasma cfDNA requires an additional extraction step in equids, and direct quantification of cfDNA from plasma is not accurate (Hobbs et al., 2024). The extraction step could limit its clinical utility in some settings, however, this step typically takes less than two hours to complete, making cfDNA analysis considerably faster than blood culture results and potentially suitable for timely clinical decision-making.

## Conclusions

Our results revealed that the NLR and plasma cfDNA concentrations are associated with bacteremia in hospitalized foals, whereas the NMR and MLR appear to have a limited relationship with blood culture status. Importantly, we generated composite models that could be utilized in the clinic to aid in the early identification of bacteremic foals, guiding clinical decision-making, and improving clinical outcomes. Future research should be aimed at determining the ability of the composite models to accurately and rapidly identify bacteremic foals at admission to a tertiary referral center.

## Supplementary Material

Supplementary Material

## Figures and Tables

**Table 1:** Comparative clinical and hematological parameters of hospitalized foals (n = 391) Positive blood culture was further separated into cohorts based on their blood culture results at admission; Gram-positive bacteremia, Gram-negative bacteremia, and polymicrobial bacteremia.

	Negative blood culture (n = 256)	Positive blood culture^#^ (n = 135)	Gram-positive blood culture^#^ (n = 56)	Gram-negative blood culture^#^ (n = 62)	Polymicrobial blood culture^#^ (n = 17)
**Age (hr)**	23 (4–48)	24^c^ (24–48)	24^b^ (12–48)	24^b^ (24–48)	48^c^ (24–72)
**HR (bpm)**	120 (96–120)	109^a^ (100–130)	112 (92–120)	104^a^ (85–123)	120 (84–140)
**RR (brpm)**	36 (28–54)	36 (24–48)	36 (27–48)	34 (25–52)	36 (32–42)
**Rectal temperature (°C)**	38.3 (37.5–38.8)	38.2 (37.5–38.8)	38.4 (38.0–38.8)	38.1 (37.1–38.9)	38.2 (36.5–39.0)
**WBC (x 10^9^/L)**	7.40 (4.55–10.4)	5.06^c^ (2.61–8.53)	5.80^a^ (3.40–8.50)	4.89^c^(2.10–9.10)	4.22^a^ (2.30–8.30)
**Neutrophils (x 10^9^/L)**	5.50 (3.00–8.57)	3.48^c^ (1.30–6.80)	4.10^b^ (1.90–6.50)	3.64^b^ (0.87–7.30)	2.10^b^ (1.10–5.80)
**Lymphocytes (x 10^9^/L)**	1.20 (0.80–1.65)	0.95^c^ (0.60–1.40)	1.03 (0.70–1.41)	0.82^c^(0.51–1.30)	1.10 (0.70–1.80)
**Monocytes (x 10^9^/L)**	0.22 (0.10–0.50)	0.18^a^ (0.10–0.32)	0.15^a^ (0.06–0.28)	0.20 (0.10–0.32)	0.18 (0.10–0.39)
**NLR**	5.0 (2.37–7.69)	3.43^b^ (1.61–6.91)	3.46 (1.81–6.83)	4.41 (0.98–7.10)	2.14^a^ (0.88–3.14)
**MLR**	0.20 (0.09–0.40)	0.19 (0.09–0.34)	0.14 (0.05–0.32)	0.25 (0.10–0.39)	0.20 (0.08–0.32)
**NMR**	0.05 (0.02–0.05)	0.06 (0.02–0.15)	0.04 (0.02–0.12)	0.06 (0.02–0.14)	0.09 (0.04–0.34)
**IgG (mg/dL)**	800 (426–1140)	506^c^ (50.0–843)	757 (393–1180)	204^c^ (0.00–725)	217^c^ (0.00–830)
**PCV (%)**	38.0 (34.0–42.0)	39.0 (34.0–43.0)	37.0 (34.0–41.0)	42.0^b^ (37.0–46.0)	33.0 (20.0–41.0)
**TS (g/dL)**	4.50 (4.00–5.30)	5.00^a^ (4.50–5.60)	5.00 (4.50–5.50)	5.00 (4.40–5.40)	5.90 (4.35–7.10)
**Glucose (mg/dL)**	117 (78.0–152)	113 (64.0–149)	123 (87.0–162)	102^b^ (39.0–122)	131 (106–170)
**L-lactate (mmol/L)**	4.10 (2.50–7.02)	3.20 (1.85–6.55)	2.95^b^(1.70–4.75)	3.80 (2.00–9.00)	3.70 (1.60–13.6)
**cfDNA (ng/mL)** *	4.47 (2.39–8.00)	7.42^b^ (4.57–15.2)	6.98^a^ (4.80–12.9)	9.27^a^ (4.10–29.0)	7.42 (2.75–42.2)

Values are reported as median and interquartile range (IQR). Statistical significance was determined using Mann-Whitney *U* test.

HR = heart rate; RR = respiratory rate; WBC = white blood cell; NLR = neutrophil-to-lymphocyte ratio; MLR = monocyte-to-lymphocyte ratio; NMR = neutrophil-to-monocyte ratio; PCV = packed cell volume; TS = total solid; cfDNA = cell-free DNA; bpm = beats per minute; brpm = breaths per minute

# significance relative to foals with a negative blood culture

a *p* < 0.05;

b *p* < 0.005;

c *p* < 0.0005

* cfDNA concentrations were not assessed in all foals. 49 foals had negative blood cultures, and 34 foals had positive blood culture. Gram-positive: 14 foals, Gram-negative: 15 foals, and polymicrobial: 5 foals

**Table 2:** Summary of multivariable logistic regression models using backward stepwise (likelihood ratio) analysis to identify independent predictors of blood culture in foals ≤ 5 days. All models were assessed for goodness-of-fit using the Hosmer–Lemeshow test.

Model 1	Exp(B)	95 % CI	Significance
cfDNA (ng/mL)	1.146	1.046–1.253	0.003
IgG (mg/dL)	0.999	0.998–1.000	0.09
NLR	0.864	0.729–1.025	0.09
WBC (x 10^9^/L)	0.862	0.737–1.010	0.066
*Constant*	*2.234*		*0.216*
**Model 2**			
Age (hr)	1.019	1.006–1.032	0.005
IgG (mg/dL)	0.999	0.845–0.986	0.001
TS (g/dL)	1.457	0.978–2.100	0.064
WBC (x 10^9^/L)	0.913	0.845–0.986	0.021
*Constant*	*0.222*		*0.133*
**Model 3**			
Age (hr)	1.016	0.999–1.033	0.06
IgG (mg/dL)	0.998	0.997-.0999	0.001
*Constant*	*1.286*		*0.545*
**Model 4**			
cfDNA (ng/mL)	1.081	1.008–1.159	0.02
IgG (mg/dL)	0.998	0.996–1.000	0.02
Neutrophils (x 10^9^/L)	0.846	0.701–1.022	0.08
*Constant*	*0.933*		*1.062*
**Model 5**			
IgG (mg/dL)	0.998	0.997–0.999	0.001
PCV (%)	1.089	1.003–1.182	0.04
*Constant*	*0.025*		*0.04*
**Model 6**			
Monocytes (x 10^9^/L)	0.076	0.011–0.555	0.011
Age (hr)	1.013	0.999–1.027	0.06
*Constant*	*0.410*		*0.011*

**Model 1**: Predictors of a positive blood culture including plasma cfDNA in the multivariable analysis. Hosmer–Lemeshow: χ^2^ = 5.63, df = 8, p = 0.689. **Model 2**: Predictors of a positive blood culture excluding plasma cfDNA in the analysis as an independent predictor. Hosmer–Lemeshow: χ^2^ = 9.21, df = 8, p = 0.325. **Model 3**: Predictors of a positive blood culture in the subset of foals meeting equine neonatal SIRS criteria (n = 152). Hosmer–Lemeshow: χ^2^ = 6.56, df = 7, p = 0.476. **Model 4**: Predictors of a Gram-negative bacteremia in the full cohort (n = 391), including cfDNA in the multivariable analysis. Hosmer–Lemeshow: χ^2^ = 9.63, df = 8, p = 0.292. **Model 5**: Predictors of a Gram-negative bacteremia excluding cfDNA as an independent predictor in the multivariable analysis. Hosmer–Lemeshow: χ^2^ = 12.56, df = 8, p = 0.128. **Model 6:**Predictors of a Gram-positive bacteremia. Hosmer–Lemeshow: χ^2^ = 5.85, df = 8, p = 0.664

Constant = the intercept, β_0_

Significance = P value < 0.1

See Table 1 for remainder of key

**Table 3:** Receiver operating characteristic (ROC) curve analysis for individual clinical and hematological variables and composite models to predict positive blood culture results in hospitalized foals. Each section of the table presents the diagnostic performance of independent predictors and multivariable models (Models 1–6) for specific outcomes: (1) positive blood culture in all hospitalized foals (n = 391), (2) positive blood culture in foals meeting the equine neonatology SIRS criteria (n = 152), and (3) more specifically, Gram-negative, Gram-positive, and polymicrobial bacteremia. Composite models were derived from multivariable logistic regression (see Table 2), and corresponding equations are listed below the table. While neutrophil and lymphocyte counts were not included in the models to avoid redundancy with the NLR, they are shown here as standalone predictors for comparative purposes.

	AUC	SE	95 % CI	Youden Index	% Sensitivity	% Specificity
***Parameters identifying positive blood culture in all hospitalized foals (n = 391)***						
**NLR**	0.582	0.03	0.521–0.644	< 3.09	49 %	68 %
**TS (g/dL)**	0.599	0.04	0.518–0.68	< 4.35	48 %	78 %
**WBC (x 10^9^/L)**	0.623	0.03	0.562–0.684	< 6.18	61 %	61 %
**cfDNA (ng/mL)**	0.670	0.06	0.539–0.789	> 4.67	75 %	55 %
**IgG (mg/dL)**	0.639	0.03	0.574–0.704	< 330	44 %	81 %
**Age (hr)**	0.650	0.02	0.591–0.700	> 23.0	76 %	50 %
**Monocytes (x 10^9^/L)**	0.590	0.04	0.515–0.665	< 0.02	63 %	53 %
**Neutrophils (x 10^9^/L)**	0.631	0.04	0.571–0.692	< 4.42	59 %	63 %
**Lymphocytes (x10^9^/L)**	0.604	0.03	0.545–0.663	< 1.05	57 %	58 %
**Model 1**	0.806	0.05	0.707–0.906	> 0.27	90 %	59 %
**Model 2**	0.746	0.05	0.665–0.826	> 0.36	70 %	74 %
***Parameters identifying positive blood culture in SIRS foals (n = 152)***						
**Age (hr)**	0.675	0.04	0.590–0.760	< 23	67 %	63 %
**IgG (mg/dL)**	0.661	0.05	0.562–0.761	< 600	61 %	76 %
**Model 3**	0.764	0.04	0.670–0.843	> 0.520	70 %	74 %
***Parameters identifying specifically Gram-negative bacteremia***						
**Neutrophils (x 10^9^/L)**	0.624	0.04	0.536–0.711	< 4.42	60 %	63 %
**PCV (%)**	0.656	0.05	0.549–0.762	> 42	47 %	77 %
**cfDNA (ng/mL)**	0.682	0.08	0.519–0.843	> 9.06	53 %	81 %
**IgG (mg/dL)**	0.730	0.03	0.651–0.870	< 330	60 %	81 %
**Model 4**	0.807	0.07	0.669–0.945	< 0.30	82 %	80 %
**Model 5**	0.780	0.05	0.674–0.884	> 0.199	81 %	76 %
***Parameters identifying specifically Gram-positive bacteremia***						
**Age (hr)**	0.632	0.04	0.559–0.705	> 23	77 %	50 %
**Monocytes (x 10^9^/L)**	0.640	0.05	0.534–0.734	< 0.19	62 %	60 %
**WBC (x 10^9^/L)**	0.610	0.04	0.529–0.690	< 6.34	56 %	61 %
**Neutrophils (x 10^9^/L)**	0.620	0.04	0.536–0.695	< 7.0	80 %	41 %
**L-lactate (mmol/L)**	0.630	0.04	0.543–0.711	< 3.25	64 %	62 %
**Model 6**	0.670	0.05	0.570–0.760	> 0.27	70 %	60 %
***Parameters identifying specifically Polymicrobial bacteremia***						
**Age (hr)**	0.764	0.04	0.687–0.841	> 26	64 %	71 %
**NLR**	0.730	0.06	0.600–0.860	< 2.66	76 %	71 %
**IgG (mg/dL)**	0.702	0.07	0.552–0.851	< 500	69 %	73 %

**Model 1:** logit(P)= 2.234 + (ln(1.146)×cfDNA)-(ln(0.999)×IgG)-(ln(0.864)×NLR)-(ln(0.862)×WBC)

**Model 2:** logit(P)= 0.222 + (ln(1.019)×age)-(ln(0.999)×IgG)-(ln(1.45)×TS)-(ln(0.913)×WBC)

**Model 3:** logit(P)= 1.286 + (ln(1.016)×age)-(ln(0.998)×IgG)

**Model 4:** logit(P)= 0.933 + (ln(1.081)×cfDNA)-(ln(0.998)×IgG)-(ln(0.846)×Neutrophils)

**Model 5:** logit(P)= 0.025 + (ln(0.998)×IgG)-(ln(1.089)×PCV)

**Model 6:** logit(P)= 0.410 + (ln(0.076)×monocytes)-(ln(1.01)×age)

**AUC** = area under the curve. **See** Table 1 for remainder of key

**Table 4:** Comparative clinical and hematological parameters between negative blood culture and positive blood culture foals meeting the equine neonatal SIRS criteria (n = 152).

	Negative blood culture (n = 83)	Positive blood culture (n = 69)
**Clinical parameters (IQR)**		
**Age (hr)**	12 (2.0–24)	24^c^ (12–48)
**HR (bpm)**	120 (100–138)	104^b^ (84–124)
**Respiratory rate (brpm)**	40.0 (30.0–60.0)	36^a^ (24.0–50.0)
**Rectal temperature (°C)**	38.2	38
**(95 % CI)**	(98.5–101.9)	(98.5–102.1)
**Hematological parameters (IQR)**		
**WBC (x 10^9^/L)**	6.00 (3.90–10.8)	4.86^a^ (2.33–7.89)
**Neutrophils (x 10^9^/L)**	4.67 (1.84–9.50)	3.10^a^ (0.96–6.40)
**Lymphocytes (x 10^9^/L)**	1.23 (0.89–1.68)	0.98^a^ (0.08–1.40)
**Monocytes (x 10^9^/L)**	0.22 (0.08–0.57)	0.16 (0.10–0.31)
**NLR**	3.62 (1.73–7.46)	3.21 (0.94–6.60)
**MLR**	0.20 (0.07–0.50)	0.18 (0.09–0.37)
**NMR**	0.04 (0.02–0.06)	0.05 (0.03–0.18)
**IgG (mg/dL)**	827 (580–1110)	400^b^ (0.00–848)
**PCV (%)**	39.0 (34.0–43.0)	40 (34.0–44.0)
**TS (g/dL)**	4.50 (4.00–5.40)	5.0 (4.40–5.40)
**Glucose (mg/dL)**	99.0 (54.0–144)	99.0 (41.0–131)
**L-lactate (mmol/L)**	6.10 (3.52–11.2)	4.4^a^ (2.25–8.65)
**cfDNA (ng/mL)** *	4.24 (2.85–11.2)	9.27^a^ (4.76–15.2)

Values are reported as median and interquartile range (IQR). Statistical significance was determined using the Mann-Whitney *U* test.

SIRS = Systemic Inflammatory Response Syndrome

**See** Table 1 for remainder of key

a *p* < 0.05;

b *p* < 0.005;

c *p* < 0.0005

* cfDNA concentrations were not assessed in all foals but a subset of 40 foals. Negative blood culture = 22 foals, Positive blood culture = 18 foals.

## Data Availability

The data that support the findings of this study are available on request from the corresponding author, R.E.T

## Appendix A. Supporting information

Supplementary data associated with this article can be found in the online version at doi:10.1016/j.tvjl.2025.106427.

## Abbreviations:

NLR: Neutrophil-to-lymphocyte ratio
NMR: Neutrophil-to-monocyte ratio
MLR: Monocyte-to-lymphocyte ratio
CfDNA: cell-free DNA
SNS: Sick non-septic
ROC: Receiver Operating Characteristic
AUC: Area Under Curve
OR: Odds Ratio
IQR: Interquartile Range

## References

[R1] Barr B, Nieman NM, 2022. Serum amyloid a as an aid in diagnosing sepsis in equine neonates. Equine Veterinary Journal 54, 922–926. 10.1111/evj.13540. 34773677

[R2] Barton MH, Morris DD, Norton N, , 1998. Hemostatic and fibrinolytic indices in neonatal foals with presumed septicemia. Journal of Veterinary Internal Medicine American College of Veterinary Internal Medicine 12, 26–35. 10.1111/j.1939-1676.1998.tb00493.x. 9503357

[R3] Bayless RL, Cooper BL, Sheats MK, 2022. Investigation of plasma cell-free DNA as a potential biomarker in horses. The Journal of Veterinary Diagnostic Investigation 34, 402–406. 10.1177/10406387221078047. 35168428 PMC9254060

[R4] Bayless RL, Cooper BL, Sheats MK, 2024. Extracted plasma Cell-Free DNA concentrations are elevated in colic patients with systemic inflammation. Veterinary Sciences 11, 1–14. 10.3390/vetsci11090427. PMC11435807 39330806

[R5] Bookbinder LC, Mani R, Carr EA, 2023. Antibiograms of field and hospital acquired equine neonatal bacterial fluid cultures in the midwestern United States: 149 samples (2007–2018). Journal of Veterinary Internal Medicine American College of Veterinary Internal Medicine 37, 1193–1200. 10.1111/jvim.16671. PMC10229355 37029453

[R6] Borchers A, Wilkins PA, Marsh PM, , 2013. Sequential L-lactate concentration in hospitalised equine neonates: a prospective multicentre study. Equine Veterinary Journal 45, 2–7. 10.1111/evj.12165. 24304396

[R7] Brewer BD, Koterba A, 1988. Development of a scoring system for the early detection of equine neonatal sepsis. Equine Veterinary Journal 20, 18–22. 10.1111/j.2042-3306.1988.tb01445.x. 3366100

[R8] Charoensappakit A, Sae-khow K, Rattanaliam P, , 2023. Cell-free DNA as diagnostic and prognostic biomarkers for adult sepsis: a systematic review and meta-analysis. Scientific Reports 13, 1–11. 10.1038/s41598-023-46663-2. 37949942 PMC10638380

[R9] Chung H, Lee JH, Jo YH, , 2019. Circulating monocyte counts and its impact on outcomes in patients with severe sepsis including septic shock. Shock Augusta Georgia 51, 423–429. 10.1097/SHK.0000000000001193. 30286035

[R10] Cohen ND, 1994. Causes of and farm management factors associated with disease and death in foals. JAVMA 204, 1644–1651. 8050947

[R11] Colmer SF, Luethy D, Abraham M, , 2021. Utility of cell-free DNA concentrations and illness severity scores to predict survival in critically ill neonatal foals. PLoS One 16, e0242635. 10.1371/journal.pone.0242635. PMC8075268 33901192

[R12] Corley KTT, Pearce G, Magdesian KG, , 2007. Bacteraemia in neonatal foals: clinicopathological differences between Gram-positive and Gram-negative infections, and single organism and mixed infections. Equine Veterinary Journal 39, 84–89. 10.2746/042516407X157585. 17228602

[R13] DeClue AE, Johnson PJ, Day JL, , 2012. Pathogen associated molecular pattern motifs from Gram-positive and Gram-negative bacteria induce different inflammatory mediator profiles in equine blood. Veterinary Journal London England 1997 192, 455–460. 10.1016/j.tvjl.2011.09.00. 21974971

[R14] Demmers S, Johannisson A, Gröndahl G, , 2001. Neutrophil functions and serum IgG in growing foals. Equine Veterinary Journal 33, 676–680. 10.2746/042516401776249327. 11770989

[R15] Djordjevic D, Rondovic G, Surbatovic M, , 2018. Neutrophil-to-Lymphocyte ratio, Monocyte-to-Lymphocyte ratio, Platelet-to-Lymphocyte ratio, and mean platelet Volume-to-Platelet count ratio as biomarkers in critically ill and injured patients: which ratio to choose to predict outcome and nature of bacteremia? Mediators of Inflammation 2018, 1–15. 10.1155/2018/3758068. PMC6079471 30116146

[R16] Draing C, Sigel S, Deininger S, , 2008. Cytokine induction by Gram-positive bacteria. Immunobiology 213, 285–296. 10.1016/j.imbio.2007.12.001. 18406374

[R17] Fingerhut L, Ohnesorge B, von Borstel M, , 2019. Neutrophil extracellular traps in the pathogenesis of equine recurrent uveitis (ERU). Cells 8, 1–22. 10.3390/cells8121528. PMC6953072 31783639

[R18] Furr M, McKenzie H, 2020. Factors associated with the risk of positive blood culture in neonatal foals presented to a referral center (2000–2014). Journal of Veterinary Internal Medicine American College of Veterinary Internal Medicine 34, 2738–2750. 10.1111/jvim.15923. PMC7694804 33044020

[R19] Gayle JM, Cohen ND, Chaffin MK, 1998. Factors associated with survival in septicemic foals: 65 cases (1988–1995). Journal of Veterinary Internal Medicine American College of Veterinary Internal Medicine 12, 140–146. 10.1111/j.1939-1676.1998.tb02109.x. 9595374

[R20] Giguère S, Weber EJ, Sanchez LC, 2017. Factors associated with outcome and gradual improvement in survival over time in 1065 equine neonates admitted to an intensive care unit. Equine Veterinary Journal 49, 45–50. 10.1111/evj.12536. 26538009

[R21] Golonka RM, Yeoh BS, Petrick JL, , 2018. Deoxyribonuclease I activity, Cell-Free DNA, and risk of liver cancer in a prospective cohort. JNCI Cancer Spectrum 2, 1–6. 10.1093/JNCICS/PKY083. PMC6383694 30815627

[R22] Hackett ES, Lunn DP, Ferris RA, , 2015. Detection of bacteraemia and host response in healthy neonatal foals. Equine Veterinary Journal 47, 405–409. 10.1111/evj.12307. 24917427

[R23] Hobbs KJ, Cooper BL, Dembek K, , 2024. Investigation of extracted plasma Cell-Free DNA as a biomarker in foals with sepsis. Veterinary Sciences 11, 1–12. 10.3390/vetsci11080346. PMC11359113 39195800

[R24] Hoeberg E, Sånge A, Saegerman C, , 2022. Serum amyloid a as a marker to detect sepsis and predict outcome in hospitalized neonatal foals. Journal of Veterinary Internal Medicine American College of Veterinary Internal Medicine 36, 2245–2253. 10.1111/jvim.16550. PMC9708439 36239317

[R25] Hollis AR, Wilkins PA, Palmer JE, , 2008. Bacteremia in equine neonatal diarrhea: a retrospective study (1990–2007). Journal of Veterinary Internal Medicine American College of Veterinary Internal Medicine 22, 1203–1209. 10.1111/j.1939-1676.2008.0152.x. 18638014

[R26] Huttunen R, Kuparinen T, Jylhävä J, , 2011. Fatal outcome in bacteremia is characterized by high plasma cell-free DNA concentration and apoptotic DNA fragmentation: a prospective cohort study. PLoS One 6, 1–8. 10.1371/journal.pone.0021700. PMC3128600 21747948

[R27] Hytychová T, Bezděková B, 2015. Retrospective evaluation of blood culture isolates and sepsis survival rate in foals in the Czech Republic: 50 cases (2011–2013). Journal of Veterinary Emergency and Critical Care 25, 660–666. 10.1111/vec.12348. 26220509

[R28] Koterba AM, Brewer BD, Tarplee FA, 1984. Clinical and clinicopathological characteristics of the septicaemic neonatal foal: review of 38 cases. Equine Veterinary Journal 16, 376–382. 10.1111/j.2042-3306.1984.tb01950.x. 6479139

[R29] Lenz M, Maiberger T, Armbrust L, , 2022. CfDNA and DNases: new biomarkers of sepsis in preterm Neonates—A pilot study. Cells 11, 1–10. 10.3390/cells11020192. PMC8774011 35053308

[R30] Letendre JA, Goggs R, 2017. Measurement of plasma cell-free DNA concentrations in dogs with sepsis, trauma, and neoplasia. Journal of Veterinary Emergency and Critical Care 27, 307–314. 10.1111/vec.12592. 28295988

[R31] Li T, Dong G, Zhang M, , 2020. Association of neutrophil-lymphocyte ratio and the presence of neonatal sepsis. Journal of Immunology Research 2020, 1–8. 10.1155/2020/7650713. PMC7728472 33344658

[R32] Li X, Chen Y, Yuan Q, , 2024. Neutrophil-to-lymphocyte ratio, monocyte-to-lymphocyte ratio, platelet-to-lymphocyte ratio associated with 28-day all-cause mortality in septic patients with coronary artery disease: a retrospective analysis of MIMIC-IV database. BMC Infectious Diseases 24, 749–759. 10.1186/s12879-024-09516-5. 39075364 PMC11288105

[R33] Liu S, Wang X, She F, 2021. Effects of Neutrophil-to-Lymphocyte ratio combined with Interleukin-6 in predicting 28-Day mortality in patients with sepsis. Frontiers in Immunology 12, 1–9. 10.3389/fimmu.2021.639735. PMC8007868 33796105

[R34] Liu VX, Fielding-Singh V, Greene JD, , 2017. The timing of early antibiotics and hospital mortality in sepsis. American Journal of Respiratory and Critical Care Medicine 196, 856–863. 10.1164/rccm.201609-1848OC. 28345952 PMC5649973

[R35] Ljungström L, Pernestig AK, Jacobsson G, , 2017. Diagnostic accuracy of procalcitonin, neutrophil-lymphocyte count ratio, C-reactive protein, and lactate in patients with suspected bacterial sepsis. PLoS One 12, 1–10. 10.1371/journal.pone.0181704. PMC5519182 28727802

[R36] Naess A, Nilssen SS, Mo R, , 2017. Role of neutrophil to lymphocyte and monocyte to lymphocyte ratios in the diagnosis of bacterial infection in patients with fever. Infection 45, 299–307. 10.1007/s15010-016-0972-1. 27995553 PMC5488068

[R37] Ng WW-S, Lam S-M, Yan W-W, , 2022. NLR, MLR, PLR and RDW to predict outcome and differentiate between viral and bacterial pneumonia in the intensive care unit. Scientific Reports 12, 1–12. 10.1038/s41598-022-20385-3. 36153405 PMC9509334

[R38] Panizzi L, Dittmer KE, Vignes M, , 2023. Plasma and synovial fluid Cell-Free DNA concentrations following induction of osteoarthritis in horses. Animals 13, 1–12. 10.3390/ani13061053. PMC10044647 36978592

[R39] PC de Jager C, TL Van Wijk P, Mathoera RB, , 2010. Lymphocytopenia and neutrophil-lymphocyte count ratio predict bacteremia better than conventional infection markers in an emergency care unit. Critical Care 14 (8), 1. 10.1186/cc9309. PMC3219299 21034463

[R40] Rackoff WR, Gonin R, Robinson C, , 1996. Predicting the risk of bacteremia in children with fever and neutropenia. Journal of Clinical Oncology Official Journal of the American Society of Clinical Oncology 14, 919–924. 10.1200/JCO.1996.14.3.919. 8622040

[R41] Raisis AL, Hodgson JL, Hodgson DR, 1996. Equine neonatal septicaemia: 24 cases. Australian Vet J 73, 137–140. 10.1111/j.1751-0813.1996.tb10006.x. 8660228

[R42] Rello J, Valenzuela-Sánchez F, Ruiz-Rodriguez M, , 2017. Sepsis: a review of advances in management. Advances in Therapy 34, 2393–2411. 10.1007/s12325-017-0622-8. 29022217 PMC5702377

[R43] Robinson JA, Allen GK, Green EM, , 1993. A prospective study of septicaemia in colostrum-deprived foals. Equine Veterinary Journal 25, 214–219. 10.1111/j.2042-3306.1993.tb02946.x. 8508750

[R44] Russell CM, Axon JE, Blishen A, , 2008. Blood culture isolates and antimicrobial sensitivities from 427 critically ill neonatal foals. Australian Vet J 86, 266–271. 10.1111/j.1751-0813.2008.00311.x. 18616477

[R45] Saenz JJ, Izura JJ, Manrique A, , 2001. Early prognosis in severe sepsis via analyzing the monocyte immunophenotype. Intensive Care Medicine 27, 970–977. 10.1007/s001340100962. 11497155

[R46] Samuels AN, Kamr AM, Reed SM, , 2024. Association of the neutrophil-lymphocyte ratio with outcome in sick hospitalized neonatal foals. Journal of Veterinary Internal Medicine American College of Veterinary Internal Medicine 38, 1196–1206. 10.1111/jvim.16995. PMC10937482 38284437

[R47] Sanchez CL, Giguere S, Lester GD, 2008. Factors associated with survival of neonatal foals with bacteremia and racing performance of surviving thoroughbreds: 423 cases (1982–2007). JAVMA 233, 1446–1452. 10.2460/javma.233.9.1446. 18980499

[R48] Scalco R, de Oliveira GN, da Rosa Curcio B, , 2023. Red blood cell distribution width to platelet ratio in neonatal foals with sepsis. Journal of Veterinary Internal Medicine American College of Veterinary Internal Medicine 37, 1552–1560. 10.1111/jvim.16793. PMC10365058 37306395

[R49] Skovbjerg S, Martner A, Hynsjö L, , 2010. Gram-positive and gram-negative bacteria induce different patterns of cytokine production in human mononuclear cells irrespective of taxonomic relatedness. Journal of Interferon Cytokine Research the Official Journal of the International Society for Interferon and Cytokine Research 30, 23–32. 10.1089/jir.2009.0033. 20028205

[R50] Stewart AJ, Hinchcliff KW, Saville WJA, , 2002. Actinobacillus sp. Bacteremia in foals: clinical signs and prognosis. Journal of Veterinary Internal Medicine American College of Veterinary Internal Medicine 16, 464–471. 10.1111/j.1939-1676.2002.tb01266.x. 12141310

[R51] Theelen MJP, Wilson WD, Byrne BA, , 2019. Initial antimicrobial treatment of foals with sepsis: do our choices make a difference? Veterinary Journal 243, 74–76. 10.1016/j.tvjl.2018.11.012. 30606442

[R52] Urosevic N, Merritt AJ, Inglis TJJ, 2022. Plasma cfDNA predictors of established bacteraemic infection. Access Microbiology 4, 1–11. 10.1099/acmi.0.000373. PMC9394668 36004363

[R53] Wagner B, Burton A, Ainsworth D, 2010. Interferon-gamma, interleukin-4 and interleukin-10 production by t helper cells reveals intact Th1 and regulatory TR1 cell activation and a delay of the Th2 cell response in equine neonates and foals. Veterinary Research 41, 1–14. 10.1051/vetres/2010019. 20374696 PMC2865874

[R54] Weber EJ, Sanchez LC, Giguère S, 2015. Re-evaluation of the sepsis score in equine neonates. Equine Veterinary Journal 47, 275–278. 10.1111/evj.12279. 24750245

[R55] Wilkins PA, Wong D, Slovis NM, , 2025. The systemic inflammatory response syndrome and predictors of infection and mortality in 1068 critically ill newborn foals. Journal of Veterinary Internal Medicine American College of Veterinary Internal Medicine 39. 10.1111/jvim.70004. PMC11911538 40091577

[R56] Wong DM, Ruby RE, Dembek KA, , 2018. Evaluation of updated sepsis scoring systems and systemic inflammatory response syndrome criteria and their association with sepsis in equine neonates. Journal of Veterinary Internal Medicine American College of Veterinary Internal Medicine 32, 1185–1193. 10.1111/jvim.15087. PMC5980351 29582480

[R57] Xia X, Wang Y, Xie M, , 2022. Elevated neutrophil - to - monocyte ratio as a prognostic marker for poor outcomes in neonatal sepsis. Heliyon 8. 10.1016/j.heliyon.2022.e11181. PMC9626927 36340000

[R58] Zahorec R, 2021. Neutrophil-to-lymphocyte ratio, past, present, and future perspectives. Bratislava Medical Journal 122, 474–488. 10.4149/BLL_2021_078. 34161115

