# Supplementary material for: The predictive ability of blood-based biomarkers to detect bacteremia in hospitalized neonatal foals
Source: Vet J. Author manuscript; Available in PMC 2026 Mar 23. (PMC13008482; doi:10.1016/j.tvjl.2025.106427)
Supplement: Supplementary Material [file NIHMS2142914-supplement-Supplementary_Material.docx]

**Supplemental Table 1.** Bacterial isolates from Blood Cultures in Hospitalized Foals (< 5 days of age) that presented to referral hospitals**.** Values are presented as absolute numbers and percentages of total isolates. Because each foal with a polymicrobial blood culture had a unique combination of bacterial isolates, these were categorized within the Gram-negative or Gram-positive groups rather than treated as a separate category.

| **Category** | **Number of isolates (%)** |
| --- | --- |
| **All Gram-negative** | **80** |
| *Escherichia sp* | 27/80 (34%) |
| *Acinetobacter sp.* | 13/80 (16%) |
| *Actinobacillus sp* | 10/80 (13%) |
| *Enterobacter sp* | 5/80 (6%) |
| *Klebsiella sp.* | 5/80 (6%) |
| Other | 20/80 (25%) |
|  |  |
| **All Gram-positive** | **70** |
| *Enterococcus sp.* | 22/70 (32%) |
| *Staphylococcus sp* | 12/70 (17%) |
| *Streptococcus sp.* | 12/70 (17%) |
| *Clostridium sp* | 5/70 (7%) |
| *Other* | 19/70 (27%) |
|  |  |
